# Measuring temperature and observing graphitization of heavy-ion-heated diamond

**DOI:** 10.1038/s41598-026-59428-4

**Published:** 2026-07-06

**Authors:** J. Lütgert, P. Hesselbach, A. Bergermann, A. J. Roy, V. Bagnoud, B. Heuser, B. Lindqvist, R. Redmer, D. Riley, G. Schaumann, M. Schörner, A. Sokolov, M. G. Stevenson, A. Tauschwitz, D. Varentsov, L. Wegert, Y. Yang, B. Zielbauer, Zs. Major, P. Neumayer, D. Kraus

**Affiliations:** 1 Institut für Physik, Universität Rostock, Albert-Einstein-Str. 23, 18059 Rostock, Germany; 2 GSI Helmholtzzentrum für Schwerionenforschung, Planckstr. 1, 64291 Darmstadt, Germany; 3 Institut für Angewandte Physik, Goethe-Universität Frankfurt am Main, Max-von-Laue-Str. 1, 60438 Frankfurt am Main, Germany; 4 SLAC National Accelerator Laboratory, Menlo Park, 94309 California, USA; 5 Helmholtz-Zentrum Dresden-Rossendorf, Bautzner Landstr. 400, 01328 Dresden, Germany; 6 Institut für Angewandte Physik, Technische Universität Darmstadt, Schlossgartenstr. 7, 64289 Darmstadt, Germany; 7 Centre for Plasma Physics, School of Mathematics and Physics, Queen’s University Belfast, University Road, BT7 1NN Belfast, United Kingdom; 8 Institut für Kernphysik, Technische Universität Darmstadt, Schlossgartenstr. 9, 64289 Darmstadt, Germany; 9 State Key Laboratory of Ultrafast Optical Science and Technology, Xi’an Institute of Optics and Precision Mechanics, Chinese Academy of Sciences, Xinxi Road 17, 710119 Xi’an, China; 10 Helmholtz Institut Jena, Fröbelstieg 3, 07743 Jena, Germany; 11 HEDI - Institute of High Energy Density Physics, Helmholtz-Zentrum Dresden-Rossendorf, Albert-Einstein-Str. 25, 18059 Rostock, Germany

**Keywords:** Materials science, Physics

## Abstract

The graphitization of diamond is of significant interest for nanodiamond synthesis from laser-shocked hydrocarbons, for its use as a detector material, and for the application in diamond anvil cells. The transition can be triggered either locally through sufficiently rapid energy deposition, or thermally at temperatures above 1800 K. We report on an experiment in which a heavy-ion beam was used to volumetrically heat monocrystalline diamond, investigating the intermediate regime between these two competing mechanisms. Our sample is probed using x-ray radiation generated from a laser-driven titanium plasma. The ratio of elastic to inelastic scattering in backward direction allows for measuring the samples’ bulk temperature. We observe good agreement between the x-ray based technique and stopping power simulations up to $\sim$ 2000 K. When increasing the heating further by fully stopping the ions in the target, we reach conditions where graphitization is predicted to occur. In this regime, elastic x-ray scattering is notably stronger than expected for pristine, heated diamond. Simultaneous x-ray diffraction measurements show a modification of the previously sharp diamond peaks in this range, which is also accompanied by changes in the optical properties of the sample. A thermally induced transition to graphite provides the most plausible explanation of the observations.

## Introduction

Despite being exceptionally hard, diamond is a metastable carbon allotrope at ambient pressure and temperature conditions. Its optical and electronic properties allow for a plethora of applications in medicine and technology. Several studies investigated the stability of diamond under heating, and its transition to graphite, which begins at about 1800 K and drastically accelerates with temperature^1,2^. In contrast to these experiments in which the graphitization occurs on the timescale of minutes, an ultrafast (fs), non-thermal transformation has been observed when using a free-electron laser depositing energies around an electronvolt per atom^3–5^. Heavy-ion beams enable unprecedented heating rates for samples of mm-size while allowing to study the interplay between temperature effects and ionizing radiation^6^. They bridge the gap between the experiments above by granting access to intermediate time scales of order of hundreds of ns and bulk temperatures of thousands of Kelvin. Such conditions are directly relevant for the cooling and expansion phase of diamonds created in laser-shock experiments^7,8^, are realized when diamonds are used in particle detectors^9^, and provide a valuable testing case for theoretical modelling of carbon in the onset of the warm dense matter (WDM) regime. Furthermore, diamond anvil cells can be combined with heavy-ion irradiation to study extreme radiation- and pressure conditions, simultaneously^10–12^. Understanding the damage thresholds of the anvils is crucial for the success of such experiments.

Recent studies on ion-heated diamond established an x-ray based technique to measure the temperature of the sample *in-situ*^13^. The previous experiment’s accuracy has been limited by notable noise on the signal, and suffered from the limited heating achieved. Temperatures were on the order of $\sim$ 1300 K, too cold to expect significant graphitization. In the present study, we report on a follow-up experiment, mitigating noise and increasing the heating. The latter was achieved by scaling the available ion numbers, improving focussing and – to reach the highest peak temperatures – by using a degrader slab to slow down the ions such that they were stopped inside the diamond target^14^. This way, we generated temperatures in excess of $\sim$ 2000 K, where graphitization is predicted.

After introducing the method to measure temperature and the experimental setup in sections X-ray based temperature measurement and Experimental setup, respectively, we present the results of the scattering diagnostic in section Measurement of diffuse elastic scattering, and compare the inferred temperatures to ion stopping power simulations. We then connect the increase in the diffuse elastic scattering to x-ray diffraction measurements and optical recordings of the sample (section XRD and imaging analysis), finding indicators for a phase transition. Its nature will be discussed in section Discussion of possible graphitization, by comparison to classical molecular dynamics and radiation damage simulations.

## X-ray based temperature measurement

The following section provides a brief overview of the applied temperature diagnostics. For a more extensive discussion, we refer the reader to prior work^13^.

Despite being an important thermodynamic state variable, temperature is demanding to measure in WDM experiments. X-ray based techniques promise insight into the bulk material, and are applicable to temperatures and samples that cannot be studied by pyrometry. For warm dense fluids, x-ray diffraction (XRD) can be leveraged as a temperature diagnostic^15,16^ by comparison with density functional theory molecular dynamics (DFT-MD) simulations.

Here, we attempt to measure the temperature of a lattice, instead. With rising temperature, the vibration of atoms in a crystalline sample increases, which in turn reduces interference effects responsible for Laue spots. This effect is well-known in the XRD community as the Debye-Waller effect, describing the reduction in intensity of the diffraction lines and increased diffuse scattering off a heated target^17,18^. However, employing XRD to obtain the temperature of diamond is challenging due to the high dynamic range required and the adequate normalization of individual shots, or the large angular range that has to be covered^19^.

Instead, spectrally resolved x-ray Thomson scattering (XRTS) can be measured, mitigating these issues.

Typically, contributions to the XRTS signal are distinguished based on the states occupied by the electron before and after the scattering process with a photon^20,21^: The full dynamic structure factor $S_{ee}^\text {tot}(k, \omega )$ can be written as

1 $$\begin{aligned} S_{ee}^\text {tot}(k, \omega ) =&|f(k) + q(k)|^2 S_{ii}(k) \times \delta (\omega )\nonumber \\ &+ S_{ee}^{\text {ff}}(k, \omega ) + S_{ee}^{\text {bf}}(k, \omega ) + S_{ee}^{\text {fb}}(k, \omega )\quad , \end{aligned}$$

where $\hbar k$ and $\hbar \omega$ are momentum and energy transfer in the scattering process, respectively, $\hbar$ is the reduced Planck constant, and $\delta$ is the delta-distribution. With *f* we denote the atomic form factor, *q* the free-electron screening cloud, $S_{ii}$ the static ion-ion structure, and $S_{ee}^{\text {ff}}(k, \omega )$, $S_{ee}^{\text {bf}}(k, \omega )$, and $S_{ee}^{\text {fb}}(k, \omega )$ are the free-free, bound-free and free-bound^22^ dynamic electron-electron structure factors, respectively. The first line of eqn. (1) describes the elastic scattering on electrons which follow the movements of the ions closely. The aforementioned, temperature-induced changes in the lattice structure are encoded in $S_{ii}(k)$, which is linked to the ion-ion pair-distribution function via a Fourier transform.

From the inelastic contributions in the second line of eqn. (1), only the bound-free scattering has to be considered for the temperature range in the scope of this study, which is still relatively low. For the same reason, *q*(*k*) can be neglected. Performing the integral over $\omega$, and using that the *L* shell ionisation energy is small compared to the Compton energy in backward scattering geometry, one can derive for the static electron-electron structure factor^13,20,23–25^

2 $$\begin{aligned} S_{ee}^{\text {tot}}(k) = |f(k)|^{2}S_{ii}(k) + \sum _{n=1}^{Z_\text {wb}}\left[ 1 - f_{n}^{2}(k)\right] \quad , \end{aligned}$$

and hence link $S_{ii}$ to the ratio of elastic to inelastic scattering $x_\text {el}/x_\text {inel}$:

3 $$\begin{aligned} S_{ii}(k) = \frac{1}{|f(k)|^{2}}\left( \sum _{n=1}^{Z_\text {wb}}\left[ 1-f_{n}^{2}(k)\right] \right) \frac{x_{\text {el}}}{x_{\text {inel}}}\quad . \end{aligned}$$

Here, $f_n$ is the contribution of an electron in state *n* to the full structure factor, which can be calculated analytically for hydrogen-like atoms^26^. The sum considers all $Z_\text {wb}$ weakly bound electrons ($Z_\text {wb} = 4$ for carbon).

$S_{ii}$ can, on the other hand, also be obtained from *ab initio* DFT-MD simulations, for a given set of state parameters such as density and temperature. By mapping the measured ratio of elastic to inelastic scattering to the ion-ion static structure factor and linking this value to the DFT-MD inputs, we can leverage the former as a diagnostic for the crystal bulk temperature.

It should be noted that a measurement of the phonon modes of the lattice in the millielectronvolt regime allows for a model-free temperature assessment via the detailed balance relation^27^. Such methods have, however, strong requirements on the x-ray source only satisfied by free-electron-lasers, and are not suitable for single-shot measurements.

## Experimental setup

**Fig. 1:**
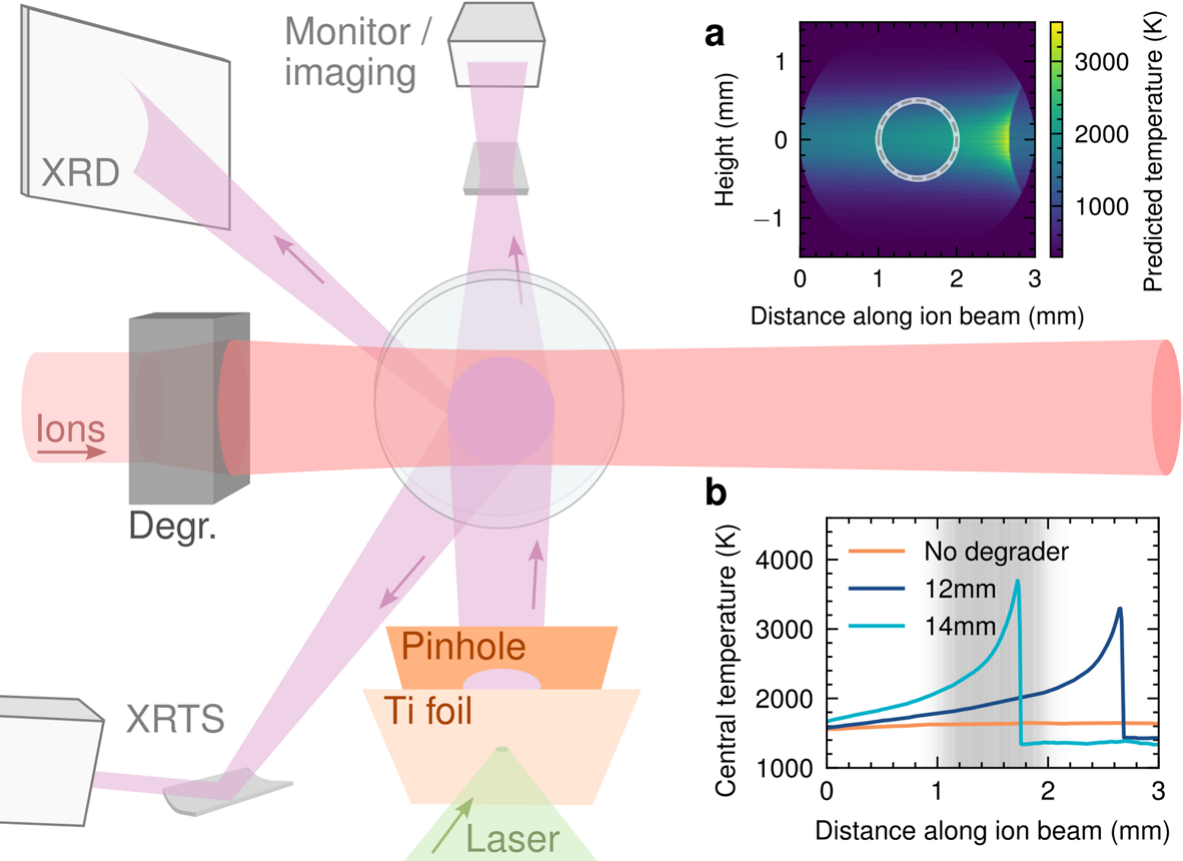
Sketch of the experimental setup. The diamond in the center was heated by a uranium-ion beam (red). To increase the energy deposition, a glassy carbon degrader (dark gray) has been used to slow down the particles. The sample was probed by laser-generated titanium $\text {He}_\alpha$ x-ray emission. A gold pinhole was used to limit the divergence of the x-rays. Inset **a**: Temperature profile for a 12 mm thick degrader, according to the stopping power simulations. **b**: Calculated temperature realized along the axis of the ion beam for different degrader thicknesses. The probed region is marked by a circle and the gray area in the insets a and b, respectively. For both insets, the calculations were performed with 4 × 10^9^ uranium ions per shot in the beamline. Note that the ion-beam is widened by the degrader slab; the extent is, however, overstated here and not to scale.

The experiment has been conducted at the high-energy, high-temperature (HHT) beamline at the GSI Helmholtzzentrum für Schwerionenforschung in Darmstadt, Germany^14^. Here, the PHELIX laser^28^ generated $\text {He}_\alpha$ x-ray emission at 4.75 keV from a titanium backlighter foil. The laser was operated at $\sim$ 160 to 180 J at 527 nm wavelength within a 2 ns pulse, and focussed to 30–40 µm spot sizes (FWHM). To ensure stable laser performance and thus a reproducible backlighter source, the experiment was limited to one shot with x-ray diagnostics per hour. The schematic setup is shown in Fig. 1. All components depicted were placed in an evacuated chamber at few 10^–4^ mbar.

GSI’s SIS18 accelerator provided a ^238^*U*^73+^ beam at 400 MeVu^–1^ with particle numbers up to 4.4 × 10^9^ per bunch. One such bunch was used to heat the sample. The pulse-length was varied between 125 and 300 ns FWHM without notable changes in integrated ion numbers. We used a fast current transformer at the target chamber entrance to measure pulse-length, particle number, and the relative timing between ion beam and probing laser. The latter could be clearly identified by its electromagnetic pulse superimposed on the current transformer signal. Summarizing all losses we find an effective beamline transmission of (0.75 ± 0.10) from the comparison with pyrometry measurements on 0.8 µm thick aluminum foils heated using similar beam parameters.

To record XRTS, we fielded a Greateyes CCD detector (GE-VAC 2048 512 BI) with a cylindrically bent HOPG (highly oriented pyrolytic graphite) crystal (radius of curvature: 50 mm, mosaicity: 0.5°, thickness: 100 µm) in von Hámos geometry^29^ at a scattering angle of 130°. To monitor the x-ray source, an identical camera was used with a planar HOPG crystal of 100 µm thickness and with a mosaicity of 0.5°. For selected shots, the monitor spectrometer was replaced by an imaging setup to optically record the sample shortly after the heating (see section XRD and imaging analysis). The spatially resolved XRD pattern was measured by a GE Healthcare BAS IP SR 2040 E imaging plate (IP) at (70.7 ± 1.5)°. As samples, we used commercially available monocrystalline CVD diamond discs of optical quality with a diameter of 3 mm. The 100 µm thick cylinders were cut so that their faces were oriented in [100] direction and rotated to position the (111) Laue peak on the XRD detector. According to the manufacturer Günter Wicke Handels-GmbH, the impurity level for nitrogen was below 1 ppm.

To increase the heating, a glassy carbon degrader (density 1.42 g cm^–3^) was fielded $\sim$ 5 cm in front of the target, reducing the kinetic energy of the ion beam. This additional component allowed us to slow down the projectiles while keeping the accelerator settings optimal for focussing the heavy-ion beam. At sufficiently low kinetic energies, projectiles deposit nearly all their energy within the sample, generating a sharp increase in the stopping power curve known as the Bragg peak (see inset b of Fig. 1). We emphasize that this phenomenon is distinct from the ‘Bragg reflections’ known in the XRD community. Tuning the degrader length allowed for shifting the Bragg peak’s position within the diamond. We fielded three degrader configurations:

1. A setup without degrader.
2. A degrader of 12 mm length, in the direction of the ion beam. With this setting, the Bragg peak was located within the sample, but not in the region probed by x-rays. We will refer to this setup as short degrader setting.
3. The long degrader configuration, using 14 mm of glassy carbon. Here, the Bragg peak is in the probed region, achieving significantly higher peak temperatures at the cost of spatial homogeneity of the heating.

Inset a of Fig. 1 shows exemplary simulations of the heating achieved with the short degrader setting. These calculations were performed using the stopping power code SRIM^30^. We accounted for the ion spot sizes (up to (0.87 × 0.48) mm^2^ without degrader for maximal ion numbers) obtained by argon fluorescence measurements^14,31^, and used ray tracing simulations to model the individual particles’ paths in the target. To calculate temperatures from the deposited energies, we used temperature-dependent heat capacities reported by Reeber and Wang^32^.

By fielding a degrader, we also alter the ion beam’s composition. FLUKA^33,34^ simulations predict a non-zero probability of splitting the uranium into lower-*Z* species, when interacting with the degrader material: About 2.4 % of the primary projectiles fragment per mm in glassy carbon. Due to the increased angular spread and lower atomic number of the fragments, their contribution to the heating of the sample is reduced, effectively lowering the number of ions for the stopping power simulations. The effect has been accounted for in the effective ion numbers and resulting temperatures presented in this manuscript (e.g., in Fig. 3). FLUKA simulations also provide an extended, effective focus size of the ion beam with degrader by increasing the beam’s divergence. We have accounted for this by convolution of the simulated energy deposition profile of a pencil beam interacting with degrader and propagated to the target with the measured distribution of the incident particles. This correction reduces the temperature by $\sim$ 200 K for high ion numbers when using the long degrader setting and has been applied to all data presented. Our calculations only consider a pristine, cold degrader, as no damage to the material was visible, even after multiple shots.

**Fig. 2:**
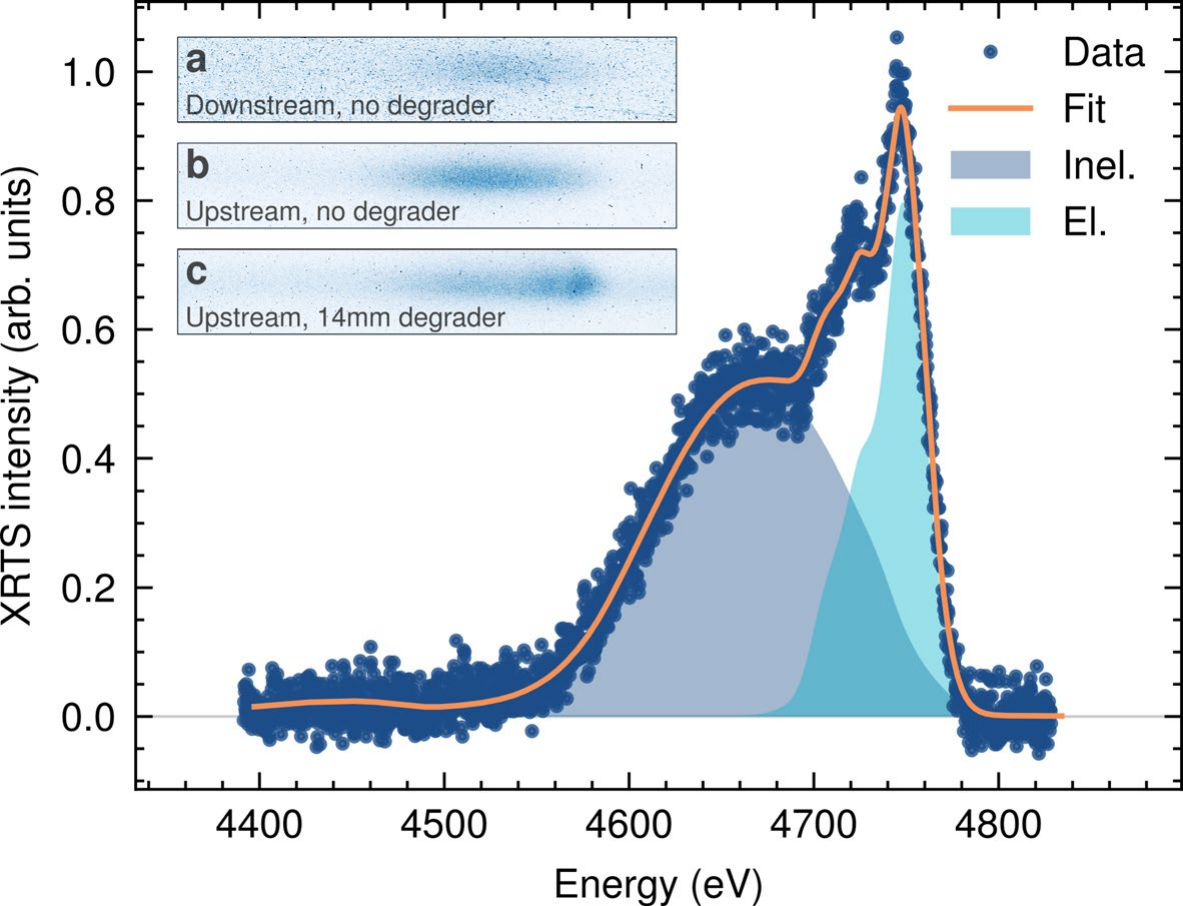
Fit of the XRTS spectrum as sum of an elastic and inelastic contribution. In the insets, the two-dimensional raw-data show the notable decrease in parasitic signal when fielding the spectrometers up- instead of downstream of the target with respect to the ion beam. The prominent traces in inset a are attributed to particles generated by the heavy-ion beam interacting with sample and degrader^14^. Inset c presents the raw data corresponding to the lineout depicted in the main figure. The elastic to inelastic scattering ratio is visibly increased when using a degrader length such that the Bragg peak is in the probed region.

Compared to previous experiments^13^, the x-ray diagnostics were fielded upstream of the target with respect to the ion beam, as we found that the particle background – caused by the ion beam interacting with the degrader and our target – is highly directional^14^. This setup-change, in combination with increased shielding, notably increased the signal-to-noise ratio by more than 3 × (see insets a and b of Fig. 2).

## Measurement of diffuse elastic scattering

The XRTS signal was fitted as a sum of two contributions: the elastic and inelastic part of the spectrum. An exemplary fit for data with substantial heating is shown in Fig. 2. Emission of the titanium plasma was modelled by the PrismSpect software^35^ to best reproduce the measured source spectrum^13^. During our campaign the performance of the PHELIX laser was reasonably consistent, producing a stable source. For shots in which the sample imager was fielded instead of the monitor spectrometer, we used typical plasma conditions of temperatures around $k_BT\approx {2}\,\textrm{keV}$ 2 keV and ion number densities of 4.2 × 10^20^cm^–3^. The elastic signal has been modelled by convolution of the source spectrum with the XRTS spectrometer’s instrument function. The inelastic contribution was inferred from a sample not irradiated by the ion beam. From the recorded cold XRTS spectrum, the elastic contribution at room-temperature ($x_\text {el} / x_\text {inel} \sim$ 1.7%, according to our DFT-MD simulations) has been subtracted. We allowed for small shifts in the dispersion between shots due to the uncertainty of target and source alignment.

**Fig. 3:**
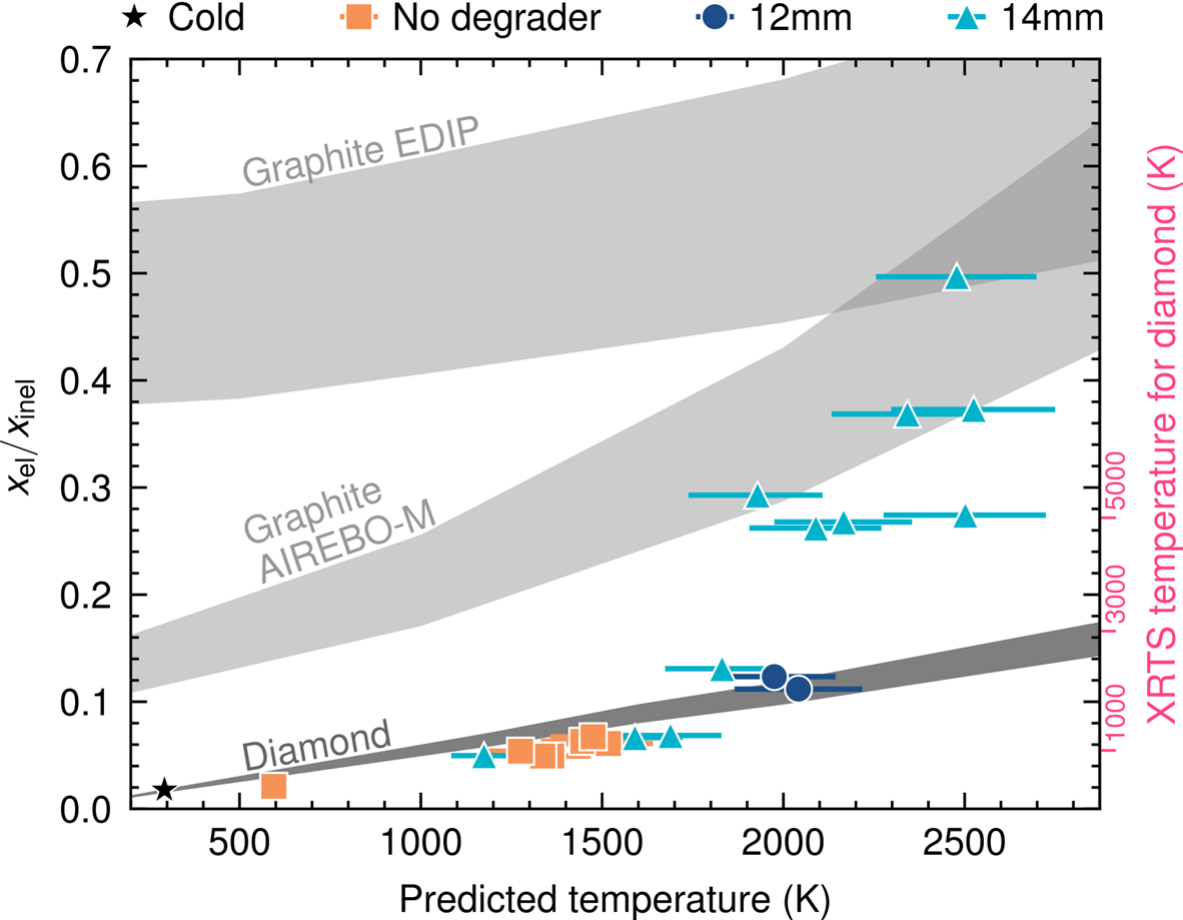
Ratio between elastic and inelastic scattering over mean sample temperature according to ion stopping power simulations. We corrected for the fragmentation of the ion beam in the degrader, the probing time, the focus size, as well as for the beamline’s transmission. Ratios for pure diamond (from DFT-MD calculations) or graphite (calculated from classical MD with the AIREBO-M potential and EDIP) are given by the gray shaded areas. The black star in the lower left shows the measured ratio for a sample at room temperature. The degrader settings are distinguished by different symbols. We observe good agreement with expected ratios for diamond up to 2000 K. Above this value, the elastic signal increases notably. The pink, secondary axis to the right indicates temperatures to which the $x_\text {el}/x_\text {inel}$ correspond to, for monocrystalline diamond. The highest ratios measured exceed reasonable values notably.

Figure 3 shows elastic to inelastic scattering ratios measured for different temperatures – achieved by varying ion number and degrader setting. We can compare these values to SRIM simulations, as they are shown in inset a of Fig. 1. For doing so, we average the two-dimensional temperature-maps over the area illuminated by x-rays, determined by ray tracing our experimental setup. These averaged temperatures are plotted on the x-axis of Fig. 3. The stated error bars account for uncertainties in the beamline transmission and the alignment of the target, but not in the kinetic energies of the projectiles.

Without degrader and for maximal ion numbers, XRTS measures a ratio $x_\text {el}/x_\text {inel}$ of 0.064 – corresponding to temperatures around (1200 ± 120) K when compared to DFT-MD calculations. Similar conditions were accomplished in a previous campaign^13^. These temperatures are slightly below the SRIM simulations, which predict (1480 ± 135)K. With the short degrader we find notably higher ratios of 0.11, corresponding to temperatures of about (2000 ± 200)K, assuming pure diamond. The stopping power calculations agree, predicting (2000 ± 190)K.

When probing the Bragg peak by using the long degrader setting, the deviation between the two methods notably increases: While below 1900 K, both temperatures coincide, the elastic scattering intensity reaches up to 50 % of the inelastic intensity at the highest ion numbers. This ratio exceeds not only the expectation for the average temperature as they are displayed in Fig. 3, but even for the predicted peak temperatures (presented in inset b of Fig. 1), when assuming a pristine diamond sample. Even at 5000 K, we would expect a scattering ratio of only 0.27. As such an interpretation of the data yields unrealistic results, we conclude that the assumption of pristine diamond is incorrect, and the XRTS signal indicates alternation of the sample in the Bragg peak. Our upscaled DFT-MD calculations (see Methods) do not capture a possible phase transition in the target, as the neural network which computes the forces on the ions during the simulation was solely trained on diamond lattices. A graphitization or amorphization of parts of the sample could, however, explain the increase of diffuse elastic scattering.

The light gray areas in Fig. 3 show the expected ratio $x_\text {el}/x_\text {inel}$ for graphite at varying temperatures. They were obtained by classical MD-simulations performed with the software package LAMMPS^36^, using the AIREBO-M interatomic potential, which predicts experimental values for the interlayer spacing of graphite reasonably well^37^, and the environment-dependent interaction potential (EDIP)^38–40^, respectively. For further details, we refer to the Methods section of this manuscript. It is apparent that XRTS measurements of graphite would show notably more diffuse elastic scattering than diamond at the same temperatures, for the conditions probed in our experiment.

In the previous study, a macroscopic fracturing of the sample was observed by XRD^13^. This result has been confirmed here, as it will be discussed in section XRD and imaging analysis. Lattice defects introduced by the diamond breaking on a macroscopic level would lead to disorder, and therefore to an increased elastic scattering – similarly as increased lattice vibrations due to temperature effects do. Rigorous modelling of this effect is challenging, and a quantitative analysis is out of the scope for this work. Exemplary calculations (shown in the Supplementary figures), however, suggest that the observed increase in $S_{ii}$ is higher than predicted with simple defect models. Furthermore, we reason that for both datasets utilizing a degrader, the Bragg peak was located within the sample. Hence, macroscopic changes to the whole sample due to temperature gradients – and with it expansion of the diamond – leading to stress and, eventually, breaking, should be similar for both setups. We note that while the probed region has a temperature of $\sim$ 2000 K using the short degrader setting, the peak energy deposition in the full sample is rather comparable to the most heated shots with a long degrader, as inset b of Fig. 1 shows. As we observe the rapid change in the diffuse scattering intensity with temperature in the center of the target discussed above, shattering of the sample appears insufficient to explain the strongly enhanced elastic peak. Graphitization or amorphization would be more locally confined than tension-induced fracture, and might therefore explain the observed steep increase better. Further insight into the samples, supporting this hypothesis, are provided by XRD measurements as well as direct imaging of the sample after ion irradiation.

## XRD and imaging analysis

**Fig. 4:**
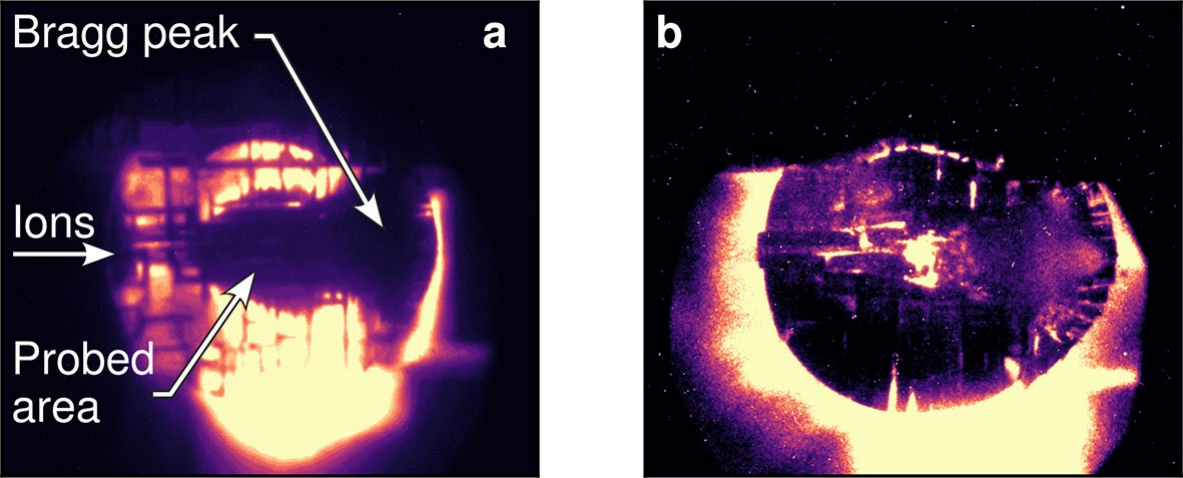
Optical imaging of the diamond after the irradiation, using (**a**) a green and (**b**) a red color filter. Due to different timing settings of the cameras, the data shows slightly different stages of the sample after irradiation, $\sim$ 3 µs after the onset of the ion beam. In the Bragg peak, the sample becomes opaque in the left recording. Further, we clearly observe the target fracturing.

For selected shots we fielded an imaging system instead of the monitor spectrometer for measuring optical images of the backlit sample. Two PCO DiCams recorded the target using a green and red color filter, respectively. To prevent damage to the cameras, we triggered them up to few microseconds after the PHELIX laser was incident onto the backlighter foil. A shot with a 12 mm degrader is displayed in Fig. 4. The green imaging system shows regions in which the optical properties of the sample change and the diamond becomes opaque. We link the area downstream of the ion beam to the Bragg peak in the target (see inset a in Fig. 1) and the other to the region where hot plasma from the backlighter, being confined by the pinhole, hits and heats the sample. This circle therefore coincides with the area probed by the x-ray flash. However, as the x-ray pulse ends way before the plasma expands to the target, the scattering volume should be solely heated by the heavy ions at the time of probing. Fracture is very prominent in these recordings, both with and without degrader (see also Supplementary figures).

**Fig. 5:**
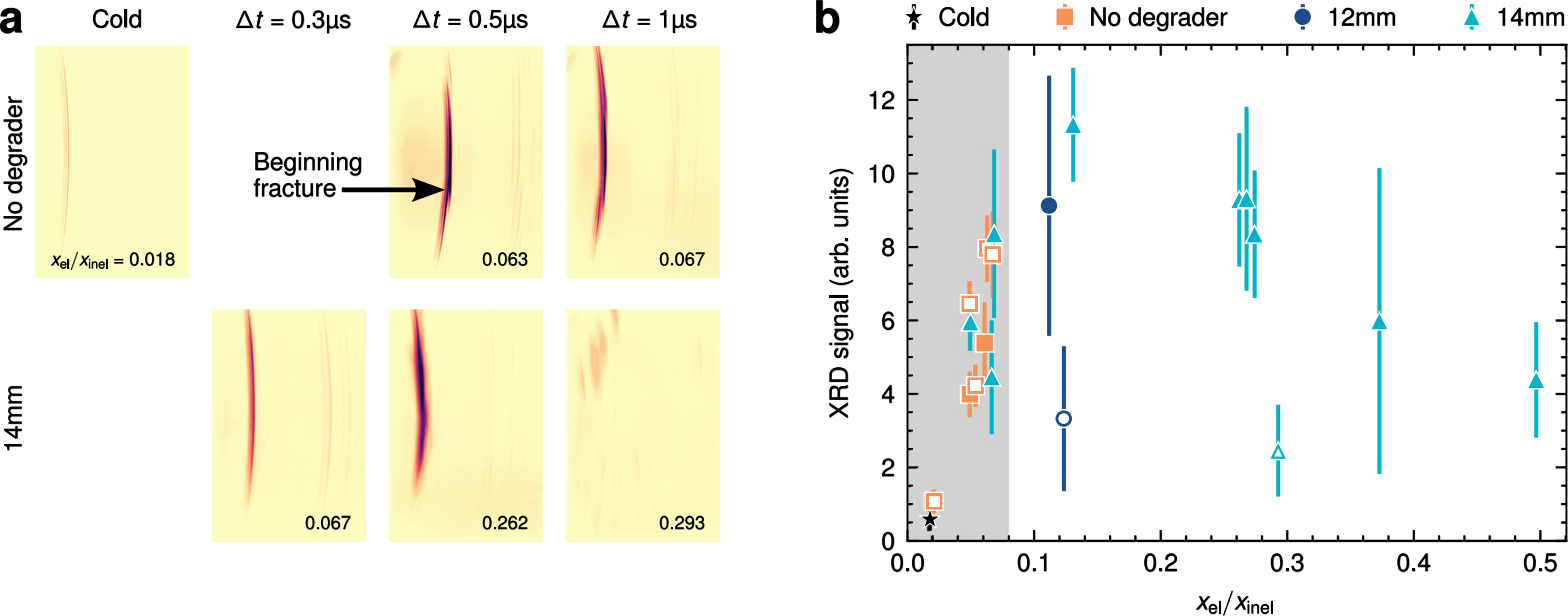
Results of the x-ray diffraction diagnostics. **a**: XRD recordings for different delays for no degrader (top) and the long degrader setting (bottom). The curvature of the signal arises from the divergence of the x-rays on the sample. Cold diffraction (upper left) shows distinct lines from the backlighter emission. Signs of fracture can be seen for driven shots – with and without degrader, albeit fracture and vanishing of the diamond signal are most prominently visible for long delays with the long degrader setting. For high-ratios of elastic to inelastic scattering (numbers in the lower-right corner), we see the diffraction lines smear out. As high and low ratios were measured for comparable fracture patterns at 0.5 µs, we conclude that shattering of the sample alone does not provide a sufficient explanation for the increase in the elastic scattering. All images are displayed on the same colorscale, showing a notable increase in the signal strengths when comparing the cold with driven shots (see right). **b**: XRD signal integrated over the whole area shown in panel a over the measured ratio of elastic to inelastic scattering. The intensity is divided by the inelastic XRTS signal to account for fluctuations in the x-ray flux. They gray area indicates where the temperatures from XRTS and ion stopping power calculations agree well, assuming a diamond sample, as shown in Fig. 3. In this area we see an increase in the XRD scattering signal (see text). At higher ratios, a reduction is observed, which could be attributed either to diffracted signal leaving the detector or to a phase transition of the sample. Shots with probing times of 1 µs or later are indicated by open symbols.

Signs for the diamond breaking apart were also clearly apparent in the diffraction images. The left side (a) of Fig. 5 compares two degrader settings and shows how the continuous lines observed for a cold sample split into multiple segments with increasing delay between heating and probing. These changes occur for several shots with varying $x_\text {el}/x_\text {inel}$ scattering ratios, and not only for those which show the highest elastic signal. We therefore reason that the macroscopic breaking of the target – while potentially contributing – does not seem to be the only explanation for our observations in the scattering diagnostic.

The effect of fracture can also be seen in an increase in the overall recorded diamond diffraction signal, which is presented in Fig. 5b. The area shaded in gray highlights events for which the ratio of elastic to inelastic scattering agrees well with the expectation based on the temperature from SRIM calculations – assuming pristine diamond. As this ratio translates to the temperature nearly linearly in this regime^13^, we observe an increase in the signal from the (111) Laue spot with heating. This effect might be unexpected, as it was discussed in section X-ray based temperature measurement that the Debye-Waller effect should reduce the scattering intensity, instead. However, due to the high Debye temperature of diamond, the reduction amounts to only 5 % when heating to 2000 K^13^. A counteracting effect could be a decreasing grain-size of the diamond sample: It is well-known that the reflectivity of a mosaic crystal is higher than that of a monocrystalline sample^41^. Practically, most materials can be described to be mosaic, with correction terms for deviations from this model which reduce the reflectivity. Such extinction effects have been reported to have strong relevance for CVD diamond^42^. The so-called primary extinction corrects for the finite size of the crystallites. For the conditions of our experiment, a reduction of grain size from 20 to 1 µm would increase the reflectivity by about 7×, due to this effect^43,44^. We remark that fracture on the considered scale seems not to impede the XRTS temperature measurement.

We also observe further features in the XRD measurements of shots with the degrader: The diamond XRD signal intensity reduces for high-ratio shots and long delays (see Fig. 5). Again, a possible explanation for this drop in the diffraction signal might be graphitization/amorphization of the sample. Another interpretation could be a fractured target, where parts rotate so that the signal leaves the active area of the IP. Additionally, with increased heating, the distinct lines from the source spectrum are blurring out. This behavior is expected, as thermal expansion of the lattice shifts the spot’s position, and temperature inhomogeneity therefore results in a wider peak.

Given the huge temperature gradients in our samples due to the degrader, the XRD data seems inconclusive beyond assessing the aforementioned macroscopic integrity of the diamond at early times. In any case, fragmentation of the target would be expected to facilitate the transition to graphite, as the resulting increase in surface area could promote the onset of the phase transformation.

## Discussion of possible graphitization

While direct evidence for graphitization is still lacking in our experiment, we nevertheless noted at several places that a graphitization or amorphization of our sample is a reasonable explanation for our experimental observations. To summarize, we saw a high ratio between elastic and inelastic scattering when the Bragg peak was in the probed region; the amount of diffuse elastic scattering cannot be explained by mere heating while keeping the monocrystalline diamond structure intact, as the required temperatures would be significantly higher than predicted by stopping-power simulations. Fracture has been discussed as a potential source, but since it can also be observed in lower-ratio shots, according to XRD, this does not seem sufficient, either. Finally, we observe the sample changing optical properties in the imaging diagnostics. Graphitization, which is predicted to occur at the temperatures reached in the Bragg peak, appears to us as the best explanation, as it would result in higher diffuse elastic XRTS as well, according to classical MD simulations.

To further investigate the phase transition and its cause, we will in the following briefly discuss thermally driven graphitization and radiation-driven graphitization/amorphization of diamond. To study the former, we performed classical MD simulations with different potentials: the reactive empirical bond order (REBO-II) potential^45^, AIREBO-M^37^ developed from it, and the EDIP^38–40^. Previous studies on the graphitization from amorphous carbon showed vast discrepancies between the potentials used^46^. The phase transition studied here yields results of similar ambiguity, highlighting the complexity of the carbon system. All potentials agree on graphitization starting from the surface at low temperatures and ambient pressures, and predict a strong directionality of the process, which is fastest along the [111] crystal direction of diamond. This finding is in-line with experimental observations^2^ and early computational studies of the phase transition^47,48^. The actual graphitization speed, however, was found to vary by about an order of magnitude between the different potentials investigated, impeding quantitative conclusions with regard to the experiment. To highlight this, we fitted the velocity of the advancing graphite layer linearly, finding (0.2 ± 0.2), (1.43 ± 0.27), and (5.57 ± 0.05) ms^–1^ for AIREBO-M, REBO-II, and EDIP, in [111] direction at 2000 K, respectively. Even the highest speeds appear to be too slow to transform the whole sample, given typical probe times $\lesssim$ 500 ns would result in $\gtrsim$ 95 % of the diamond remaining intact. The [100] direction, along which our samples were cut, proves all the more stable, with only the EDIP suggesting graphitization at 2000 K, and with reduced velocities of (1.82 ± 0.14) ms^–1^. When considering the notably higher peak temperature of the Bragg peak, the graphitization velocity increases for all potentials. While the transformation speed remains comparatively slow for the AIREBO-M potential, EDIP and REBO-II predict that notable parts of the diamond could graphitize during the time between heating and probing. Along the fasted [111] direction, REBO-II estimates $\sim$ 80 % of the target to graphitize at 3000 K; the EDIP even calculates a complete phase transition. The latter potential furthermore predicts a slow onset of a volumetric graphitization, at temperatures around $\gtrsim$ 1800 K, on top of the surface effect, at ambient pressures. This behavior can, however, not be observed when using the AIREBO-M or REBO-II potentials up to 3000 K.

Additionally, stress-induced fracture, as discussed above, could result in smaller diamond splinters, graphitizing a volume faster. Given these uncertainties and the vast difference in the behavior of the potentials tested, thermal graphitization might suffice as the driving mechanism of the phase transition. This would be indicated most by the EDIP, that has been benchmarked to describe the $sp^2$/$sp^3$transition properties well^7,47^.

The stability of diamond under radiation has been investigated mainly due to its application in particle detectors^9,49^. In these studies, the integrated dose typically exceeds this experiment by orders of magnitude, but the sample is irradiated over a much longer time. Track formation within diamond due to radiation damage has only been recently observed by Amekura *et al*^50^. In their work, they find that tracks can only occur for slow particles with huge stopping power, i.e., for heavy projectiles. While uranium in its Bragg peak might be barely above the threshold for track formation, according to MD simulations, experimental evidence has only been obtained when using accelerated fullerene molecules^50^. Tracks from uranium have not been detected in ambient diamond^11^, and while radiation damage in the uranium Bragg peak itself has been observed, it was limited to length-scales much smaller than micrometers^51^.

Ion irradiation studies typically observe a (partial) amorphization. Fairchild *et al.* attribute this failing of the diamond lattice to the density-change caused by damage in form of vacancies due to the ion beam^52^. This is supported by diamonds showing amorphization under mechanical stress^53^. In their work, the threshold for amorphization is given by 3 × 10^22^ vac/cm^3^, with other studies finding up to three times higher or lower values^52^. SRIM simulations of the damage in our samples suggest vacancy densities more than three orders of magnitude lower than this threshold. It should be noted that these simulations consider a cold target, and neglect any dynamic effects. The interplay between heating and local energy deposition by the ions on the generation of vacancies in diamond is, to our knowledge, not exhaustively explored.

The experimental setup presented should be well suited to shed light into the mechanism of the observed phase transition if an increase in ion flux would allow for reaching temperatures around 1800 to 2000 K without a degrader. For now, a thermally driven graphitization appears to be the most plausible explanation for the data at hand, as it would be compatible with (some) MD simulations – and there is no indication for a dramatic loss of radiation hardness of heated diamond.

## Conclusions and outlook

We performed XRTS and XRD measurements on ion-heated, monocrystalline diamond targets. In comparison to previous studies^13^, we notably increased the signal-to-noise ratio by improving shielding and fielding the x-ray diagnostics upstream of the sample, relative to the ion beam. These improvements directly support the simulations performed by Hesselbach *et al.*^14^, experimentally.

The use of a degrader slab enabled us to increase the heating of the sample notably by moving the Bragg peak into the probed area. While the temperature measurements from XRTS agree well with ion stopping power calculations below $\sim$ 2000 K, both methods differ for the most-heated shots. The most plausible explanation for this discrepancy seems to be a graphitization of the sample. However, direct insight into nature of the phase transition cannot be obtained with the current setup. Analysis of the sample post irradiation, e.g., using the techniques of electron microscopy or Raman spectroscopy, could provide a complementary view. Recovery of the sample would require altering the setup, and could be pursued in future work.

Finding that diamond breaks in our setup has direct implications for ion-irradiated and ion-heated diamond anvil cells. While ions remain a good option to heat the whole sample volume and trigger extreme chemistry and phase transitions via ionisation, forgoing the highest achievable energy deposition rate might be required to ensure the stability of the anvils.

Our experiment shows the current capabilities at GSI’s HHT beamline. We have demonstrated the ability of our platform to measure bulk temperatures and explore phase transitions, on large spatial and temporal scales, by using a degrader. The presented approach can be extended to study further sample types and different heating rates. The study at hand gives valuable insights for fielding x-ray based diagnostics at a heavy-ion accelerator facility, showing the importance of simple setup considerations to overcome unique sources of noise. Such effects are even more relevant for the increased ion numbers envisioned for the FAIR facility^6^. An increase in the ion flux would allow heating to comparable temperatures without fully stopping the ions, giving direct insight into the mechanism behind the phase transition as we could decouple heating from the energy deposition of an individual particle. Further, this would create more homogeneous sample conditions, and lift constraints on the ion stopping and fragmentation simulations, allowing for a more robust analysis.

## Methods

### DFT-MD simulations

The DFT-MD simulations of diamond shown in this work were performed with the Vienna *ab initio* simulation package (VASP)^54–56^. The electronic and ionic parts were decoupled by the Born-Oppenheimer approximation. At fixed ion positions, the electronic problem was solved in the finite temperature DFT approach^57^ using a projector augmented wave pseudopotential (labeled PAW_PBE C_h)^58,59^ and the Perdew-Burke-Ernzerhof functional^60^ for the exchange-correlation contribution. A 4x4x4 grid, centered at the $\Gamma$-point was employed for the *k*-space sampling, and a plane wave cutoff energy of 1000 eV was used. To scale the simulations to 8000 ions, the forces and energies predicted by DFT were learned by a Behler-Parrinello high-dimensional neural network potential (HDNNP)^61^ implemented in the n2p2 software package^62–64^. The symmetry functions were chosen according to the scheme presented by Imbalzano *et al.*^65^ with a maximum cutoff radius of 4 Å. The trained HDNNP was used to compute the electronic forces on the ions in the MD simulation within the LAMMPS software package^36^. See Schörner *et al.*^66^ for further details. The temperature control in all MD simulations was performed by a Nosé-Hoover thermostat^67,68^.

### Classical MD simulations

Classical MD simulations employing varying potentials were used to assess the graphite static structure factors, and graphitization velocities of diamond samples. For the former application, boxes with 1.1 × 10^6^ to 1.5 × 10^6^ atoms arranged in a graphite structure (COD entry 9000046) were simulated at constant temperature and pressures with periodic boundary conditions. Structure factors have been averaged over different snapshots and simulations with varying atom numbers to reduce box size effects. Simulations with a different initial structure (COD entry 9011577) yielded comparable results.

The graphitization speed was addressed by initializing a diamond structure with a temperature dependent lattice constant^69^ where one of the directions – the surface – allowed for expansion, while the others were set to periodic boundary conditions. The “Identify diamond structure” contribution^70^ to OVITO^71^ was used to calculate the fraction of atoms remaining in the initial lattice at all time steps. This quantity has been used for calculating the speed of the graphitization process that started from both free surfaces of the diamond.

## Supplementary Information

Supplementary Information.

## Data Availability

The data that support the findings of this study are available from the corresponding authors upon reasonable request.

## References

[1] Evans, T. & James, P. F. Proceedings of the Royal Society of London. *Series A. Math. Phys. Sci.* **277**, 260. 10.1098/rspa.1964.0020 (1964).

[2] Davies, G. & Evans, T. Proceedings of the Royal Society of London. *A. Math. Phys. Sci.* **328**, 413. 10.1098/rspa.1972.0086 (1972).

[3] Heimann, P. et al. Non-thermal structural transformation of diamond driven by x-rays. *Struct. Dyn.* **10**, 054502. 10.1063/4.0000193 (2023). 37901681 10.1063/4.0000193 PMC10613085

[4] Lipp, V., Tkachenko, V., Stransky, M., Aradi, B. Frauenheim, T. & Ziaja, B. Density functional tight binding approach utilized to study X-ray-induced transitions in solid materials. *Sci. Rep.* **12**, 1551. 10.1038/s41598-022-04775-1 (2022). 35091574 10.1038/s41598-022-04775-1 PMC8799736

[5] Lipp, V. et al. Exploring x-ray irradiation conditions for triggering ultrafast diamond graphitization. *Phys. Rev. B* **111**, 024103. 10.1103/physrevb.111.024103 (2025).

[6] Schoenberg, K. et al. High-energy-density-science capabilities at the Facility for Antiproton and Ion Research *Phys. Plasmas* **27**, 043103. 10.1063/1.5134846 (2020).

[7] Heuser, B. et al. Release dynamics of nanodiamonds created by laser-driven shock-compression of polyethylene terephthalate *Sci. Rep.* **14**, 12239. 10.1038/s41598-024-62367-7 (2024). 10.1038/s41598-024-62367-7 PMC11133328 38806565

[8] Safaltın, Ş & Gürmen, S. Molecular dynamics simulation of size, temperature, heating and cooling rates on structural formation of Ag-Cu-Ni ternary nanoparticles (Ag34-Cu33-Ni33) *Comput. Mater. Sci.* **183**, 109842. 10.1016/j.commatsci.2020.109842 (2020).

[9] Trischuk, W. et al. Diamond Particle Detectors for High Energy Physics. *Nucl. Part. Phys. Proc.* **273–275**, 1023. 10.1016/j.nuclphysbps.2015.09.160 (2016).

[10] Lang, M. et al. Energy loss of 50-GeV uranium ions in natural diamond. *Appl. Phys. A* **80**, 691. 10.1007/s00339-004-3104-1 (2005).

[11] Glasmacher, U. et al. Phase Transitions in Solids Stimulated by Simultaneous Exposure to High Pressure and Relativistic Heavy Ions *Phys. Rev. Lett.* **96**, 195701. 10.1103/physrevlett.96.195701 (2006). 10.1103/PhysRevLett.96.195701 16803109

[12] Lang, M. et al. Combined high pressure and heavy-ion irradiation: a novel approach. *J. Synchrotron Radiat.* **16**, 773. 10.1107/s0909049509034384 (2009). 19844013 10.1107/S0909049509034384

[13] Lütgert, J. et al. Temperature and structure measurements of heavy-ion-heated diamond using in situ X-ray diagnostics. *Matter Radiat. Extrem.* **9**, 047802. 10.1063/5.0203005 (2024).

[14] Hesselbach, P. et al. Platform for laser-driven X-ray diagnostics of heavy-ion heated extreme states of matter. *Matter Radiat. Extrem.* **10**, 017803. 10.1063/5.0233548 (2025).

[15] Lütgert, J. et al. Measuring the structure and equation of state of polyethylene terephthalate at megabar pressures *Sci. Rep.* **11**, 12883. 10.1038/s41598-021-91769-0 (2021). 10.1038/s41598-021-91769-0 PMC8213800 34145307

[16] Kraus, D. et al. The structure of liquid carbon elucidated by in situ X-ray diffraction. *Nature* **642**, 351. 10.1038/s41586-025-09035-6 (2025). 40399671 10.1038/s41586-025-09035-6 PMC12158773

[17] Murphy, W. J., Higginbotham, A., Wark, J. S. & Park, N. Molecular dynamics simulations of the Debye-Waller effect in shocked copper. *Phys. Rev. B* **78**, 014109. 10.1103/physrevb.78.014109 (2008).

[18] Schuster, A. K. et al. Measurement of diamond nucleation rates from hydrocarbons at conditions comparable to the interiors of icy giant planets. *Phys. Rev. B* **101**, 054301. 10.1103/physrevb.101.054301 (2020).

[19] Descamps, A. et al. Evidence for phonon hardening in laser-excited gold using x-ray diffraction at a hard x-ray free electron laser. *Sci. Adv.* **10**, eadh5272. 10.1126/sciadv.adh5272 (2024). 38335288 10.1126/sciadv.adh5272 PMC10857355

[20] Chihara, J. Interaction of photons with plasmas and liquid metals - photoabsorption and scattering *J. Phys. Condens. Matter* **12**, 231. 10.1088/0953-8984/12/3/303 (2000).

[21] Gregori, G., Glenzer, S. H., Rozmus, W., Lee, R. W. & Landen, O. L. Theoretical model of x-ray scattering as a dense matter probe *Phys. Rev. E* **67**, 026412. 10.1103/physreve.67.026412 (2003). 10.1103/PhysRevE.67.026412 12636827

[22] Böhme, M. P. et al., Evidence of free-bound transitions in warm dense matter and their impact on equation-of-state measurements. *Contrib. Plasma Phys.* e70149. 10.1002/ctpp.70149 (2026).

[23] James, R. W. *The optical principles of the diffraction of x-rays*, edition repr ed., The crystalline state, Vol. 2 (George Bell & Sons, Ltd., London, 1962).

[24] Pelka, A. et al. Ultrafast Melting of Carbon Induced by Intense Proton Beams. *Phys. Rev. Lett.* **105**, 265701. 10.1103/physrevlett.105.265701 (2010). 21231678 10.1103/PhysRevLett.105.265701

[25] Kraus, D. Probing the complex ion structure in liquid carbon at 100 GPa. *Phys. Rev. Lett.* **111**, 255501. 10.1103/physrevlett.111.255501 (2013). 24483747 10.1103/PhysRevLett.111.255501

[26] Pauling, L. & Sherman, J. *Screening constants for Many-electron Atoms. The calculation and interpretation of X-ray Term values, and the Calculation of Atomic Scattering Factors* (Akademische Verlagsgesllschaft M.B.H, Leipzig, 1932).

[27] Descamps, A. et al. An approach for the measurement of the bulk temperature of single crystal diamond using an X-ray free electron laser. *Sci. Rep.* **10**, 14564. 10.1038/s41598-020-71350-x (2020). 32884061 10.1038/s41598-020-71350-x PMC7471281

[28] Major, Zs. et al. High-energy laser facility PHELIX at GSI: Latest advances and extended capabilities. *High Power Laser Sci. Eng.* **12**, 1. 10.1017/hpl.2024.17 (2024).

[29] von Hámos, L. Röntgenspektroskopie und Abbildung mittels gekrümmter Kristallreflektoren. I. Geometrisch‐optische Betrachtungen. *Ann. Phys.* **409**, 716. 10.1002/andp.19334090608 (1933).

[30] Ziegler, J. F., Ziegler, M. & Biersack, J. SRIM – The stopping and range of ions in matter (2010). *Nucl. Instrum. Methods Phys. Res. B: Beam Inter* **268**, 1818. 10.1016/j.nimb.2010.02.091 (2010).

[31] Varentsov, D. et al. Transverse Optical Diagnostics for Intense Focused Heavy Ion Beams. *Contrib. Plasma Phys.* **48**, 586. 10.1002/ctpp.200810092 (2008).

[32] Reeber, R. R. & Wang, K. Thermal expansion, molar volume and specific heat of diamond from 0 to 3000K. *J. Electron. Mater.* **25**, 63. 10.1007/bf02666175 (1996).

[33] Ferrari, A., Sala, P. R., Fasso, A. & Ranft, J. *FLUKA: A Multi-particle Transport Code*. 10.2172/877507 (2005).

[34] Böhlen, T. et al. The FLUKA Code: Developments and Challenges for High Energy and Medical Applications. *Nucl. Data Sheets* **120**, 211. 10.1016/j.nds.2014.07.049 (2014).

[35] MacFarlane, J. et al. Simulation of the ionization dynamics of aluminum irradiated by intense short-pulse lasers. In: *Proc. Inertial Fusion and Sciences Applications*, 457 (2003).

[36] Thompson, A. P. et al. LAMMPS - a flexible simulation tool for particle-based materials modeling at the atomic, meso, and continuum scales. *Comput. Phys. Commun.* **271**, 108171. 10.1016/j.cpc.2021.108171 (2022).

[37] O’Connor, T. C., Andzelm, J. & Robbins, M. O. AIREBO-M: A reactive model for hydrocarbons at extreme pressures. *J. Chem. Phys.* **142**, 024903. 10.1063/1.4905549 (2015). 25591383 10.1063/1.4905549

[38] Marks, N. A. Generalizing the environment-dependent interaction potential for carbon. *Phys. Rev. B* **63**, 035401. 10.1103/physrevb.63.035401 (2000).

[39] Lucas, G., Bertolus, M. & Pizzagalli, L. An environment-dependent interatomic potential for silicon carbide: calculation of bulk properties, high-pressure phases, point and extended defects, and amorphous structures. *J. Phys. Condens. Matter* **22**, 035802. 10.1088/0953-8984/22/3/035802 (2010). 21386297 10.1088/0953-8984/22/3/035802

[40] Jiang, C., Morgan, D. & Szlufarska, I. Carbon tri-interstitial defect: A model for the D center. *Phys. Rev. B* **86**, 144118. 10.1103/physrevb.86.144118 (2012).

[41] Als-Nielsen, J. & McMorrow, D. *Elements of Modern X-ray Physics* (Wiley, 2011). 10.1002/9781119998365.

[42] Stoupin, S., Ruff, J. P. C., Krawczyk, T. & Finkelstein, K. D. X-ray reflectivity of chemically vapor-deposited diamond single crystals in the Laue geometry. *Acta Crystallogr. A Found. Adv.* **74**, 567. 10.1107/s2053273318009439 (2018). 30182943 10.1107/S2053273318009439

[43] Sabine, T. M. The flow of radiation in a real crystal. In *International Tables for Crystallography*, Vol. C - Mathematical, physical and chemical tables, third ed. (ed. Prince E.) 609–616 (Springer, Dordrecht, 2004).

[44] Straasø, T., Dippel, A.-C., Becker, J. & Als-Nielsen, J. Model-independent structure factors from powder X-ray diffraction: A novel approach. *J. Synchrotron Radiat.* **21**, 119. 10.1107/s1600577513028269 (2014). 24365925 10.1107/S1600577513028269

[45] Brenner, D. W. et al. A second-generation reactive empirical bond order (REBO) potential energy expression for hydrocarbons *J. Phys. Condens. Matter* **14**, 783. 10.1088/0953-8984/14/4/312 (2002).

[46] de Tomas, C., Suarez-Martinez, I. & Marks, N. A. Graphitization of amorphous carbons: A comparative study of interatomic potentials. *Carbon* **109**, 681. 10.1016/j.carbon.2016.08.024 (2016).

[47] De Vita, A., Galli, G., Canning, A. & Car, R. A microscopic model for surface-induced diamond-to-graphite transitions. *Nature* **379**, 523. 10.1038/379523a0 (1996).

[48] Adiga, S. P., Curtiss, L. A. & Gruen, D. M. Molecular dynamics simulations of nanodiamond graphitization. In *Nanodiamonds: Applications in Biology and Nanoscale Medicine* (ed. Ho, D.) 35–54. 10.1007/978-1-4419-0531-4_2 (Springer US, Boston, 2010).

[49] Bhattacharya, A., Grotjohn, T. A. & Stolz, A. Degradation of single crystal diamond detectors in swift heavy ion beams. *Diamond Relat. Mater.* **70**, 124. 10.1016/j.diamond.2016.10.009 (2016).

[50] Amekura, H. et al. Latent ion tracks were finally observed in diamond. *Nat. Commun.* **15**, 1786. 10.1038/s41467-024-45934-4 (2024). 38413643 10.1038/s41467-024-45934-4 PMC10899563

[51] Hickey, D., Jones, K. & Elliman, R. Amorphization and graphitization of single-crystal diamond - A transmission electron microscopy study. *Diamond Relat. Mater.* **18**, 1353. 10.1016/j.diamond.2009.08.012 (2009).

[52] Fairchild, B. A. et al. Mechanism for the amorphisation of diamond. *Adv. Mater.* **24**, 2024–2029. 10.1002/adma.201104511 (2012). 22419269 10.1002/adma.201104511

[53] Pastewka, L., Moser, S., Gumbsch, P. & Moseler, M. Anisotropic mechanical amorphization drives wear in diamond. *Nat. Mater. ***10**, 34 10.1038/nmat2902 (2011). 21113152 10.1038/nmat2902

[54] Kresse, G. & Hafner J. Ab initio molecular dynamics for liquid metals. *Phys. Rev. B* **47**, 558(R). 10.1103/physrevb.47.558 (1993). 10.1103/physrevb.47.558 10004490

[55] Kresse, G. & Hafner, J. Ab initio molecular-dynamics simulation of the liquid-metal–amorphous-semiconductor transition in germanium. *Phys. Rev. B* **49**, 14251. 10.1103/physrevb.49.14251 (1994). 10.1103/physrevb.49.14251 10010505

[56] Kresse, G. & Furthmüller, J. Efficient iterative schemes for ab initio total-energy calculations using a plane-wave basis set. *Phys. Rev. B* **54**, 11169. 10.1103/physrevb.54.11169 (1996). 10.1103/physrevb.54.11169 9984901

[57] Mermin, N. D. Thermal properties of the inhomogeneous electron gas. *Phys. Rev.* **137**, A1441. 10.1103/physrev.137.a1441 (1965).

[58] Blöchl, P. E. Projector augmented-wave method. *Phys. Rev. B* **50**, 17953. 10.1103/PhysRevB.50.17953 (1994). 10.1103/physrevb.50.17953 9976227

[59] Kresse, G. & Joubert, D. From ultrasoft pseudopotentials to the projector augmented-wave method. *Phys. Rev. B* **59**, 1758. 10.1103/PhysRevB.59.1758 (1999).

[60] Perdew, J. P., Burke, K. & Ernzerhof, M. Generalized gradient approximation made simple. *Phys. Rev. Lett.* **77**, 3865. 10.1103/PhysRevLett.77.3865 (1996). 10062328 10.1103/PhysRevLett.77.3865

[61] Behler, J. & Parrinello, M. Generalized neural-network representation of high-dimensional potential-energy surfaces. *Phys. Rev. Lett.* **98**, 146401. 10.1103/PhysRevLett.98.146401 (2007). 17501293 10.1103/PhysRevLett.98.146401

[62] Morawietz, T., Singraber, A., Dellago, C. & Behler, J. How van der Waals interactions determine the unique properties of water. *PNAS* **113**, 8368. 10.1073/pnas.1602375113 (2016). 27402761 10.1073/pnas.1602375113 PMC4968748

[63] Singraber, A., Morawietz, T., Behler, J. & Dellago, C. Parallel multistream training of high-dimensional neural network potentials. *J. Chem. Theory Comput.* **15**, 3075. 10.1021/acs.jctc.8b01092 (2019). 30995035 10.1021/acs.jctc.8b01092

[64] Singraber, A. n2p2 - a neural network potential package (2021).

[65] Imbalzano, G. et al. Automatic selection of atomic fingerprints and reference configurations for machine-learning potentials. *J. Chem. Phys.* **148**, 241730. 10.1063/1.5024611 (2018). 29960368 10.1063/1.5024611

[66] Schörner, M., Rüter, H. R., French, M. & Redmer, R. Extending *ab initio* simulations for the ion-ion structure factor of warm dense aluminum to the hydrodynamic limit using neural network potentials. *Phys. Rev. B* **105**, 174310. 10.1103/PhysRevB.105.174310 (2022).

[67] Nosé, S. A unified formulation of the constant temperature molecular dynamics methods. *J. Chem. Phys.* **81**, 511. 10.1063/1.447334 (1984).

[68] Hoover, W. G. Canonical dynamics: Equilibrium phase-space distributions. *Phys. Rev. A* **31**, 1695. 10.1103/PhysRevA.31.1695 (1985). 10.1103/physreva.31.1695 9895674

[69] Jacobson, P. & Stoupin, S. Thermal expansion coefficient of diamond in a wide temperature range. *Diamond Relat. Mater.* **97**, 107469. 10.1016/j.diamond.2019.107469 (2019).

[70] Maras, E., Trushin, O., Stukowski, A., Ala-Nissila, T. & Jónsson, H. Global transition path search for dislocation formation in Ge on Si(001). *Comput. Phys. Commun.* **205**, 13. 10.1016/j.cpc.2016.04.001 (2016).

[71] Stukowski, A. Visualization and analysis of atomistic simulation data with OVITO–the Open Visualization Tool. *Model. Simul. Mater. Sci. Eng.* **18**, 015012. 10.1088/0965-0393/18/1/015012 (2010).

