# Supplementary material for: Measuring temperature and observing graphitization of heavy-ion-heated diamond
Source: Sci Rep. 2026 Jul 6;16:20758. doi: 10.1038/s41598-026-59428-4 (PMC13338392; doi:10.1038/s41598-026-59428-4)
Supplement: Supplementary file 1 — Supplementary Information. [file 41598_2026_59428_MOESM1_ESM.pdf]

Supplementary Figures to

Measuring Temperature and Observing Graphitization of Heavy-Ion-Heated Diamond

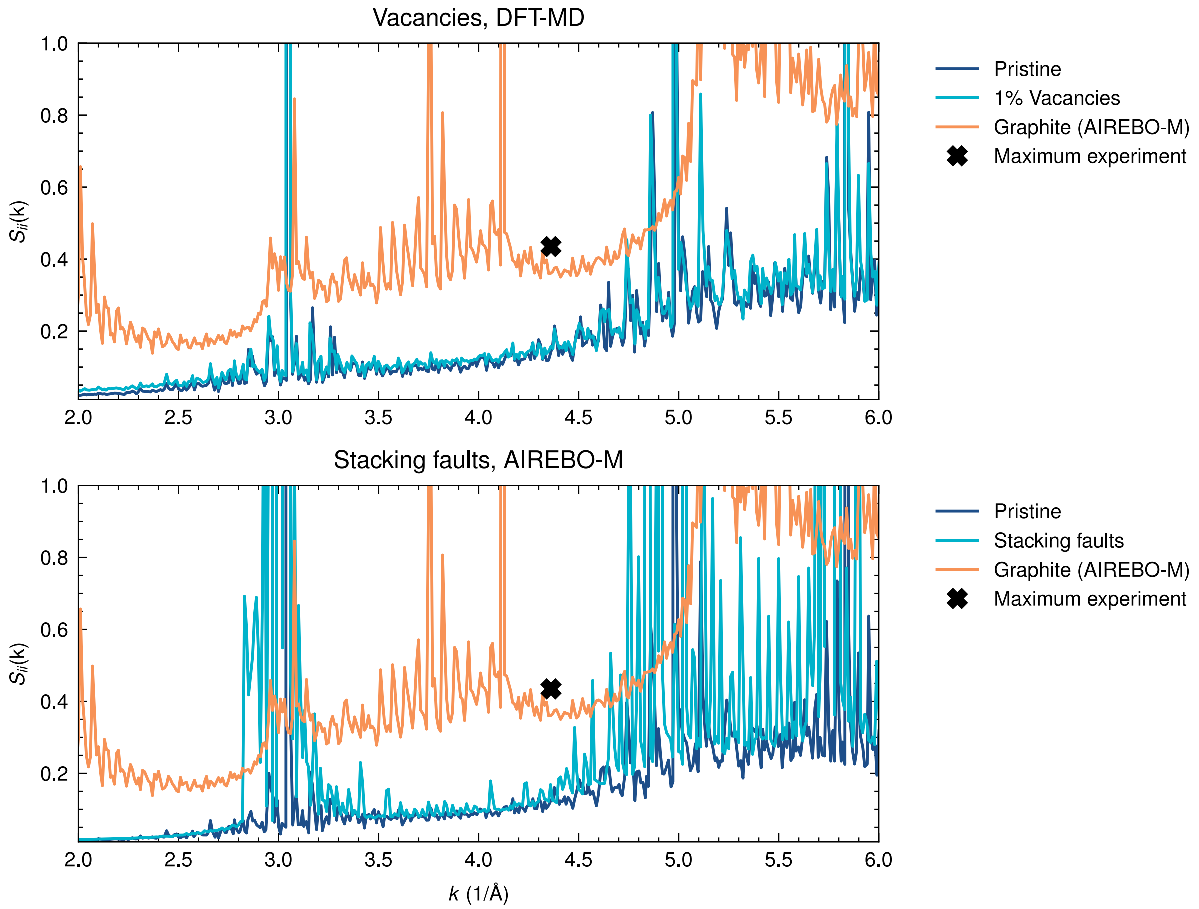

Figure S1. Simulations estimating the effect of defects on the diamond ion-ion static structure factor. All simulations were performed at 3,000 K and with a diamond lattice constant of 3.567 Å. Top: DFT-MD simulations comparing a pristine sample with a simulation where 1 % of the atoms were removed after initializing the lattice. While we observe an effect for low k and low T, such vacancies result in small deviations for the conditions probed. Bottom: Effect of stacking faults along the [111] lattice direction with layer thicknesses of few nm compared to monocrystalline diamond. In contrast to the calculations in the upper panel, we here simulated the diamond with classical MD, employing the AIREBO-M potential. While effects at and around the Laue spots are apparent, the height of the signal between peaks is less sensitive to the defect. In both figures, $S_{ii}(k)$ for graphite at 3,000 K is shown, corresponding better to the maximal value that was measured in the experiment.

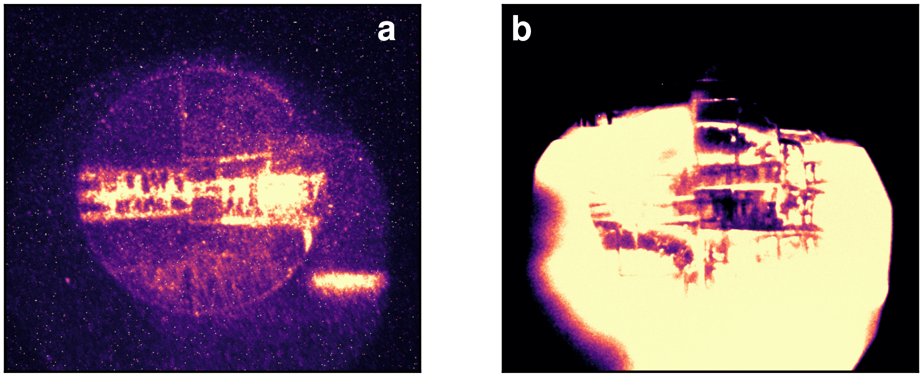

Figure S2. Optical imaging for a shot without degrader, using (a) a green and (b) a red color filter. Even without the Bragg peak in the target, we observe the diamond shatter. Inset a also shows a ghost image, slightly shifted down and right.

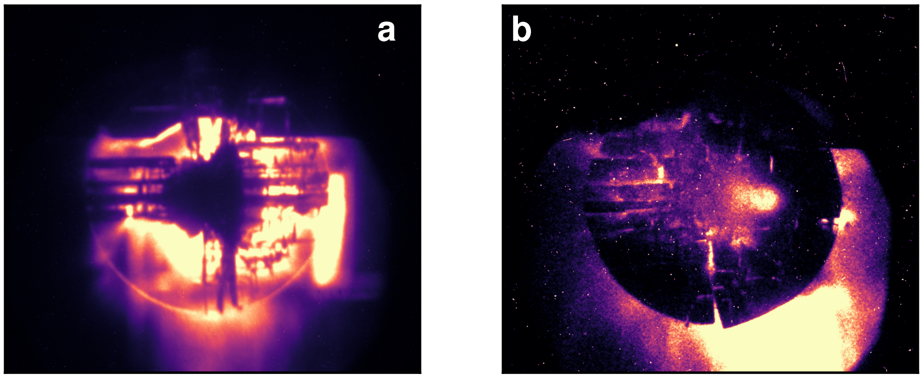

Figure S3. Optical imaging for a shot with the long degrader, using (a) a green and (b) a red color filter. The Bragg peak lies withing the region probed.
